# Centre-of-Mass Confounds Contribute to Familiar Size Stroop Effects with Boger and Firestone’s ‘Visual Anagrams’

**DOI:** 10.5334/joc.500

**Published:** 2026-04-28

**Authors:** Gregory Davis

**Affiliations:** 1 Department of Psychology, University of Cambridge, United Kingdom

**Keywords:** Semantics, Vision, Visual perception

## Abstract

‘Visual Anagrams’ are images that are recognised as one familiar object from one angle, but another object, when rotated (Geng et al., 2024). Boger and Firestone’s (2025) ingenious application of them uses examples that appear to be a large object from one angle, a small object at another. They report Familiar-Size Stroop Effects using those stimuli, claiming they must have reflected high-level recognition processes, as each visual anagram comprised identical visual features, just rotated. However, rotating visual anagrams introduces a range of *subtle potential confounds*. For example, in B&F’s stimuli, anagrams perceived as small objects had lower centres of mass (CoMs), those perceived as large objects, higher CoMs. CoM confounds might therefore account for those Stroop Effects. To assess this, a new experiment used stimuli with similar overall shapes (and CoM confounds) to B&F’s stimuli but designed to be less recognisable. The new experiment revealed Stroop effects, smaller than those observed by B&F, but consistent with a partial contribution to B&F’s findings. These results highlight important considerations when using visual anagrams in future research.

## Introduction

A perennial problem for the science of visual cognition is how to control images’ lower-level features (luminance, oriented edges, textures and hues) while manipulating their higher-level properties (e.g., the category of object they depict). Typically, when stimuli differ in terms of their higher-level properties, they also differ in terms of low-level features (see, e.g., Long, et al., 2016; Hagen et al., 2024); these may cause the behavioural or physiological effects an author attributes to high-level processes. This Methods Note examines a recent advance in solving this problem (Boger & Firestone, 2025; hereon, ‘B&F’), which introduced a new method of controlling low-level features while changing the perceived category of stimuli. While this Methods Note highlights *general* issues when using their techniques, the *particular* claims here concern only B&F’s stimuli and only their effects of these on the ‘Familiar Size Stroop Effect’.

### The Familiar Size Stroop Effect

**Figure 1:**
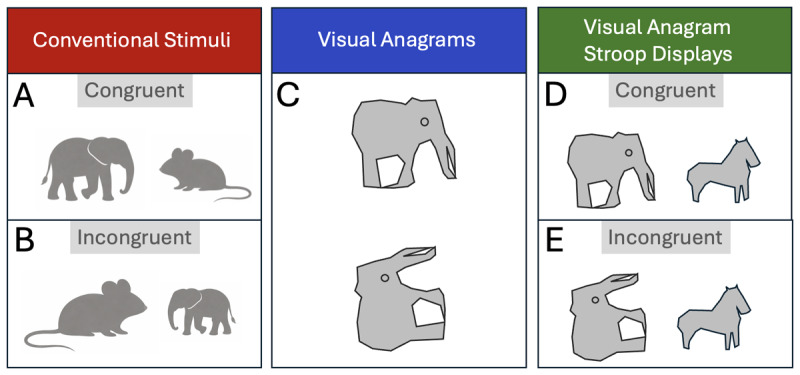
Left Panels: Cartooned conventional Familiar Size Stroop Task Displays. **(A)** ‘Congruent’ display – the larger image depicts a larger object than the smaller image, **(B)** ‘Incongruent’ display – the larger image depicts a smaller real-world object than the smaller image. Centre Panel **(C)**: Cartoon of a B&F ‘visual anagram’ – the top and bottom images are identical, other than being rotated by 90 degrees from one another, yet the perceived category of the stimulus shifts from category (elephant to rabbit). Right Panels: **(D)** When the larger visual stimulus, the visual anagram from C, is perceived as an elephant – a larger real-world object than the horse – this is a congruent trial display. **(E)** When it is perceived as a rabbit, this becomes an Incongruent trial display (a rabbit is a smaller real-world object than a horse). These displays can therefore manipulate perceived categories of the depicted objects, while controlling many (rotationally-invariant) low-level features.

B&F’s primary experiments – those showing effects of their techniques on human performance – examined the ‘Familiar Size Stroop Effect’ (e.g., Konkle & Oliva, 2012). This effect is typically observed when participants are presented with two images of real-world familiar objects and must rapidly judge which *image* is larger (or smaller). In ‘Congruent’ displays the *larger* image depicts a *larger* real-world object than the smaller image does. In Figure 1A, for example, the *larger* image depicts an elephant, a *larger* real-world, familiar object than that depicted by the smaller image (of a mouse). That is, task-relevant information that the left image is larger (than the right), and the task-irrelevant information that it depicts a larger object (than the right image does), are congruent. Figure 1B, however, shows an ‘Incongruent’ display, in which the *larger* image depicts a *smaller* real-world object (a mouse) than that depicted by the smaller image (an elephant); the task-relevant size information about the left-image being larger, and the task-irrelevant information about the depicted object’s size being small, are *incongruent*. Though participants are instructed to judge the size of *the images in the display*, ignoring the sizes of the depicted objects, response times (RTs) are typically slowed in Incongruent trials relative to Congruent trials (e.g., Konkle & Oliva, 2012; Brody et al., 2023). This slowing is often assumed to reflect automatic processing of depicted objects’ (task-irrelevant) real-world sizes, which in Incongruent trials, mismatches image-size information relevant to the task (e.g., Konkle & Oliva, 2012).

The Familiar Size Stroop Effect, then, is a behavioural effect often ascribed to high-level categorisation (e.g., Konkle & Oliva, 2012; Brody et al., 2023). However, in some previous studies, low-level confounds may explain part of the effect (see e.g., Long & Konkle, 2017; Hagen et al., 2024). For example, when images from an experiment are manipulated to retain lower-level features yet make them unrecognisable (e.g., Long et al., 2016; Freeman & Simoncelli, 2011), small Stroop Effects are *still observed* (e.g., Long & Konkle, 2017; Hagen et al., 2024). This finding may reflect associations between lower-level visual features and types of objects (e.g., rectilinear features with buildings), which could yield Stroop effects in the absence of explicit recognition (e.g., Hagen et al., 2024). On the other hand, such associations likely do not explain the Familiar Size Stroop Effect in its entirety. There is also indirect evidence of pure higher-level influences under some conditions. For example, Brody et al. (2023) found that when images of toys (e.g., toy cars, toy animals) appeared to affect the Familiar Size Stroop Effect as though they were the much larger objects they represent, rather than small toys, except when two toys are presented in the same display. Such apparent context-sensitive, symbolic associations suggest that at least part of the Familiar Size Stroop Effect reflects high-level processes.

Efforts to measure high-level and low-level contributions to the Familiar Size Stroop Effect are hampered by imperfect control of low-level features when manipulating high-level properties of stimuli. To meet this challenge, B&F introduced the use of ‘visual anagrams’ (Geng et al., 2024) – AI-generated images that appear to be a large familiar real-world object when viewed at one orientation, but a small object when viewed at a second orientation (when the image is rotated). To illustrate, consider B&F’s striking visual anagram that is perceived as a rabbit at one angle of rotation, but as an elephant at the other angle. A pared-down version of this is provided in Figure 1C (see B&F, for the original). Rotating the stimulus switches perception of the object’s category from rabbit to elephant. That is, by taking a single stimulus, and simply rotating it, an experimenter might manipulate the perceived category of an image (a high-level property) retaining *exactly* the same low-level features (rotation aside).

Figures 1D and 1E show pared-down, illustrative versions of typical displays in B&F’s experiments. Note the larger image (on the left) in both cases is the visual anagram from Figure 1C. However, it is perceived as an elephant in Figure 1D but (when rotated) a rabbit in Figure 1E. This manipulation renders Figure 1D a Congruent display: the *larger* image on the left depicts a *larger* object (an elephant) than the smaller image (of a horse, on the right). Yet, Figure 1E, comprising the same features (other than rotation of the left image) is an Incongruent display: the *larger* image now depicts a *smaller* object (a rabbit) than the right image (a horse).

By constructing Congruent and Incongruent displays from “identical” features, B&F were therefore able to measure Familiar Size Stroop Effects without the low-level feature confounds that have hindered interpretation of other studies. This approach of transforming images in the Familiar Size Stroop Task has been tried before (e.g., Hagen et al., 2024, used inversion to render images less recognisable), but B&F’s stimuli are a novel and powerful means of *switching* the perceived category of a stimulus while controlling its (rotation-invariant) low-level features. Their innovation will likely advance stimulus control in the study of high-level vision.

### Limitations of Visual Anagrams: General and Specific

Nonetheless, rotating a stimulus *does* alter its visual features. B&F acknowledge this, but explicit, detailed consideration may inform our choices of stimuli and tasks when using visual anagrams. For example, rotating a stimulus necessarily influences the orientation of stimulus elements, for example with respect to cardinal orientations preferentially coded by human vision (e.g., Girshick et al., 2011). Second, the shape’s height and breadth will often be altered, as will processing of vertical-axis symmetry, shading and convexity (e.g., Wenderoth, 1994; Ramachandran, 1988). Third, where the image spans upper and lower hemifields or left and right hemifields, it may be differentially affected by processing asymmetries (e.g., Levine & McAnany, 2005). Finally, irrespective of such asymmetries, a shape’s *centre of mass* will typically also change. These potential issues with stimulus rotation as a means of controlling stimulus features are *general* in that they likely relating to many cases of rotation. However, this Methods Note focuses on one *particular* illustrative case: a centre of mass confound in Boger & Firestone’s (2025) Stroop experiments.

**Figure 2:**
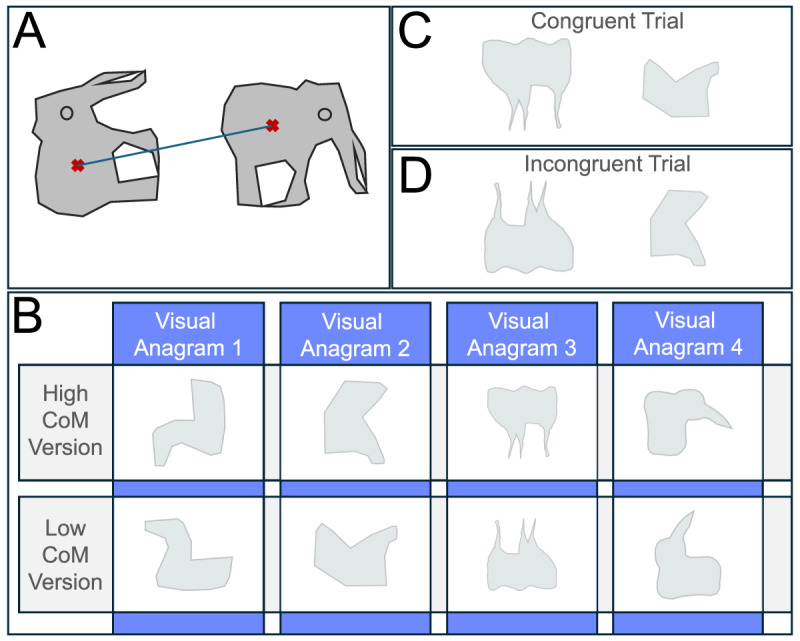
**(A)** In B&F’s stimulus set, rotating each stimulus such that it shifts from being perceived as a small object to a large object is always associated with a rise in its CoM. **(B)** Shape pairs (visual ‘anagrams’) in the current experiment: these were designed to be silhouettes with similar CoMs to B&F’s stimuli, but to be less easily categorised as familiar objects, **(C)** Congruent trial display in Experiment 1: the larger image has a higher CoM than the smaller image, **(D)** Incongruent trial display: the larger image has a lower CoM than the smaller image.

In B&F’s visual anagrams, the rotation required to shift participants’ recognition of the stimulus from a small object to a large one *always* raised the image’s centre-of-mass (CoM). To illustrate, consider the rabbit/elephant visual anagram in Figure 2A: as rotation alters recognition from smaller object (rabbit) to larger (elephant), the object’s (or image’s) centre of mass is also raised (approximated by a red ‘x’ in Figure 2A). As this affected *all of B&F’s visual anagrams*, it was a potentially serious confound. Could it, rather than object recognition feasibly have accounted for their findings? Perhaps. It has recently been claimed that objects with higher CoM are perceived to be larger (Pisu et al., 2025).

### A New Experiment

To investigate whether the centre-of-mass confounds could account for B&F’s findings (partly or wholly) in their Familiar Size Stroop Task, a new experiment was conducted. This used stimuli based on the outlines of four of five of B&F’s ‘visual anagrams’ (rabbit-elephant, duck-horse, butterfly-bear, mouse-cow); their fifth was not used, due to poor recognition (see methods). These new stimuli (Figure 2B; also referred to as ‘visual anagrams’, for simplicity) had internal features removed, leaving only silhouettes, so as to minimise recognition of them as familiar objects. Yet, as they (approximately) shared outlines with B&F’s stimuli, the CoM confounds present in B&F’s stimuli should be approximately retained here. On this basis, any Stroop effects due to CoM alone in B&F’s experiments should also be observed here, while any due to shape categorisation effects should be absent.

### ‘Congruent’ and ‘Incongruent’ Trials in the Current Work

Recall that in B&F’s displays, ‘Congruent’ trials arose when the larger image was recognised as *larger* familiar object (also with a higher CoM) than the smaller image, ‘Incongruent’ trials arose when the larger image was recognised as a smaller object (also with a lower CoM), than did the smaller image. In the current work, the aim was to reduce recognition of the shapes, leaving just the CoM component differing between Congruent and Incongruent Displays. Accordingly, ‘Congruent’ trials would now simply be those in which the larger image had a higher CoM than the smaller image, ‘Incongruent’ trials, those in which the larger image had a lower CoM than the smaller image. If CoM confounds in B&F’s Stroop Task were responsible for their effect, similar effects should also be expected here. Conversely, if CoM confounds did not contribute to B&F’s effect, none would be expected here. Given the two potentially-informative outcomes of these (pre-registered) hypotheses we planned to use a one-tailed, Bayesian t-test to assess evidence for the Alternative Hypothesis (presence of a Stroop Effect) versus for the Null Hypothesis (absence of a Stroop Effect) with these new stimuli.

### Follow-up Analysis: Minimising Categorisation influences

A key vulnerability of this approach was that, despite the author’s efforts to minimise participants’ recognition of the stimuli as familiar objects, it was still expected to arise. To assess and mitigate impacts of recognition on the Stroop effects measured here, a post-experiment questionnaire asked participants to report any categorisation of each version of each visual anagram used. These responses were then used to repeat the primary analysis but to exclude any trials involving a visual anagram that might feasibly have yielded a Familiar Size Stroop Effect on the basis of this recognition.

To anticipate findings here, participants *did* typically (mis)identify the visual anagrams as resembling various objects, but did not typically do so in way that might feasibly have yielded a Familiar Size Stroop Effect (and hence comprised the current experiment). If the Higher CoM version of any visual anagram was categorised as the same object as, or a *smaller* object than, the low CoM version, that would be expected to minimise any Stroop effect. Only if the High CoM version was categorised as a larger object than the Low CoM version would that recognition be expected to yield a Stroop Effect. As an extra safeguard, any visual anagram where only one version was categorised (identified as a familiar object) were also removed (as it was unclear what effect would be expected in such cases). This should have removed any trials with visual anagrams for which a participant’s explicit recognition of the shape could have feasibly contributed to the Stroop effect.

Note that exclusion or inclusion of trials sometimes depended on judging which of two category labels (taken from participants’ responses to the post-experiment questionnaire) was the larger object. Typically, the author relied on reasonably objective judgments about the real-world sizes of categorised shapes. A shape identified as a dog can confidently be assumed to be larger than a tooth, an elephant shape larger than a cat. One difficult case concerned a bird and rabbit comparison (rabbit was considered to be larger). This was complicated further for 3 participants, who explicitly labelled one image ‘kiwi’, the other ‘rabbit’: as this could not feasibly have impacted the current results (affecting so few participants), it was not investigated further.

## Experiment 1

### Methods

#### Preregistration and Open Science

Hypotheses, analyses and interpretation (https://aspredicted.org/7g36-vdkp.pdf) were pre-registered. Materials and Data are available at https://osf.io/unxjk/overview?view_only=a04f41d0c9da4b28bc95e8cde23df26e.

In extra analyses, the pre-registration stated: ‘We shall also ask participants if any of the shapes presented had resembled real-world objects to them, while they performed the task. We may explore whether excluding stimuli or participants with high levels of recognition substantially impacts our results.’. Here, the extra analysis excluded trials containing categorised stimuli for each participant, selected to retain adequate post-hoc power (0.86) for that analysis.

#### Participants

Ethics Permission was received from the University of Cambridge Psychology Ethics Committee (#9894.306). Thirty-two participants were recruited via the Prolific Site (www.prolific.com), aged 18–35, resident in the United Kingdom and whose first language was English (on the basis of their declared characteristics). One participant, whose mean response time was greater than 2.5 standard deviations above the group mean was excluded from analysis (as per the pre-registered analysis plan).

#### Stimuli and Apparatus

Participants used their own laptops or Desktop PCs, running Gorilla online experiment software (Anwyl-Irvine et al., 2020). Figure 2B shows the four object-pairs used in the experiment. The top shape in each panel shows the rotated version of each shape in which the centre of mass was higher; these shapes are described here as the ‘High’ (CoM) stimuli. The bottom shape in each panel shows the ‘Low’ (CoM) version of each shape (a rotated version of each shape). If shapes’ CoMs affect the Familiar Size Stroop Task as expected, ‘High’ CoM stimuli should affect performance in the same way as familiar, large objects (e.g., elephant) and the Low CoM stimuli should affect performance in the same way as familiar, small objects (e.g., mouse). Note, B&F used 5 such stimulus pairs; the fifth (lorry, lighter) was excluded here as informal survey suggested that the lighter was not readily recognised (by members of the author’s lab).

#### Procedure

**Figure 3:**
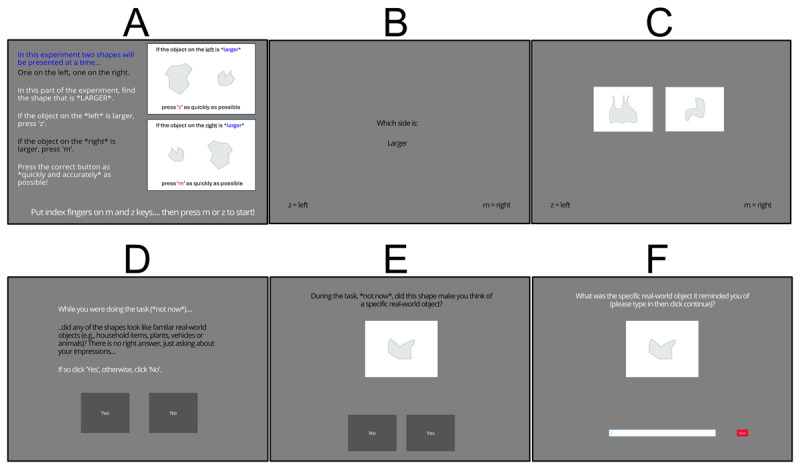
Displays from Experiment 1. **A)** Instructions Display, **B)** Initial task-reminder that initiated each trial, **C)** Stroop Task Display, **D)** Initial Display asking Participants whether they had recognised any of the shapes as familiar objects, **E)** Example display asking about each of the shapes individually, in turn, and **F)** Display with text box allowing participants to enter labels of objects they perceived the shapes to resemble, following a ‘Yes’ response to the display in E.

Following completion of the electronic informed consent process (reading of the Participant Information Sheet and completion of the Informed Consent Form), participants received task instructions (Figure 3A). There were four blocks of 48 trials each, the first two of which asked the participant to detect which of two presented shapes was larger, the latter two, which of two presented shapes was smaller (for half of participants, chosen at random, this order was reversed). At the beginning of the first two blocks and at the beginning of the second two blocks, 6 extra practice trials were presented, using simple triangle, pentagon and circle stimuli. The remaining trials used the stimuli illustrated in Figure 2B; see Figure 3C for example display.

Each trial started with the centrally presented instruction “Which side is larger” (or, in half of blocks of trials, “Which side is smaller”; 800 ms), and a reminder of the keys to respond ‘left’ (press ‘z’) and ‘right’ (press ‘m’; Figure 3B). The Shapes Display was then presented (Figure 3C; till response), in which one shape was presented on the left, one on the right. Correct responses elicited the feedback ‘Correct!’ in green text for 400 ms, Incorrect responses, the text ‘Incorrect – your accuracy is NN%’) in red text for 1500 ms. In all cases, one large shape was presented and one small shape (the small shapes were 60% the size of the large shapes along each dimension), one on the left, one on the right and two shapes were never drawn from the same pair of shapes (i.e., never just rotated versions of one another).

The participant’s task was to press the correct key as quickly and accurately as possible, to indicate whether the left or right image was larger (or smaller, according to instruction). As described in the Introduction, half of trials were Congruent and half, Incongruent. If CoM (High versus Low) impacts the Familiar Size Stroop task using stimuli very approximately similar to B&F’s, slower RTs in the Incongruent Trials than the Congruent Trials should be expected. If, however, CoM does not influence the Familiar Size Stroop Task, no such effect should be observed once shapes are not recognised as particular real-world objects. Self-paced breaks separated each block of 48 trials and when the participant switched from selecting (after 2 blocks) the larger object to selecting the smaller object (or vice versa), a brief instruction set indicated this.

Following completion of the Stroop Task trials, participants were asked whether any of the stimuli had resembled, to them, during the trials, familiar objects. If they answered yes (by clicking on one of two screen buttons; see Figure 3D), they were asked this questions about each of the possible shapes in turn, and for those shapes they responded ‘yes’ to (again by clicking on a screen button, see Figure 3E), entering the name of that object into a free-text box (Figure 3F).

### Results

RTs and Accuracy for Congruent and Incongruent Trials were analysed using Matlab and JASP (JASP Team, 2025) to ensure the same values were calculated. One participant, whose RTs were more than 2.5 standard deviations above the mean was excluded from analysis. This exclusion did not affect conclusions here – the participant showed a large Stroop Effect. Single RTs greater than 3 standard deviations were also trimmed from RTs, again following the preregistered plan.

**Figure 4:**
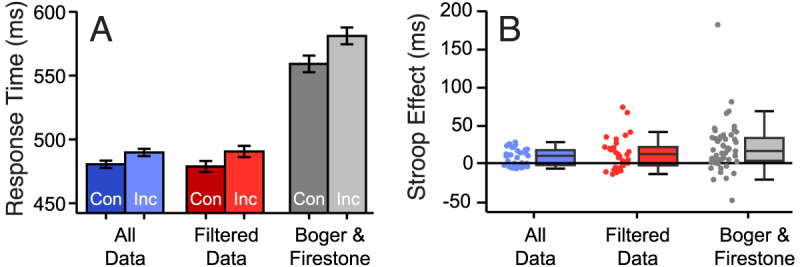
Stroop Effects in Experiment 1. **A)** Mean RTs (95% CI bars for paired designs) for Congruent and Incongruent Trials, when All trials were included (Blue Bars), only trials in which explicit recognition probably did not explain the effect (Red bars, see text), and B&F’s Experiment 1, for comparison. **B)** Box plots of Stroop Effects (Incongruent RT – Incongruent RT) with individual participants’ difference scores; format as for Figure 4A.

Figure 4A’s left (blue) columns, “All Data”, plot mean RTs for Congruent and Incongruent trials in Experiment 1 for the primary analysis (all trials included). Figure 4B’s left (blue) columns, “All Data” provide a box plot of the Stroop Effect (Incongruent RT – Congruent RT) for those conditions and individual participants’ scores. A one-tailed, paired, Bayesian t-test (JZS prior, Cauchy width 0.707) then compared evidence for a Stroop-Effect (RTs on Incongruent trials being greater than on Congruent trials; H_1_) versus no Stroop Effect (H_0_). This found very strong evidence for the presence rather than the absence of a Stroop Effect: BF_+0_ = 785.618, d = 0.835, CI 95% = [0.378, 1.195]. To check that interpretation of the RT effect was not complicated by changes in accuracy across conditions, a corresponding Bayesian t-test examined *accuracy* in Congruent trial and Incongruent trial responses. This found moderate evidence for the *absence* rather than presence of a Stroop Effect: BF_+0_ = 0.127, d = 0.112, CI 95% = [0.004, 0.319].

Figure 4A centre (red) columns, “Filtered Data”, plot mean RTs again, but now only for trials on which no shape was presented that the participant had categorised in a way that might give rise to a Familiar Size Stroop Effect. These remaining trials (after problematic classification trials for each participant had been excluded) are referred to as ‘Filtered Data’ trials, here. Trials were removed from each participant’s data according to the scheme set out in the Introduction (Supplementary Table S1 details individual decisions made around each participant’s reported labels). These exclusions completely removed data from a further two participants. Figure 4B’s (red) centre columns, “Filtered Data”, similarly plot the Stroop Effect (RT differences) for trials with only Filtered Data trials. As on the complete dataset, a Bayesian t-test compared evidence for a Stroop-Effect (H_1_) versus no Stroop Effect (H_0_), still finding ‘strong’ evidence for the presence rather than absence of a Stroop Effect: BF_+0_ = 16.1989, d = 0.565, CI 95% = [0.150, 0.909].

Results from the primary analysis then, appeared to be confirmed by the follow-up analysis. There was clear evidence of a small 9 milliseconds Stroop effect whether or not potentially problematic trials were removed. While this effect might seem small (versus e.g., B&F’s E1: 21 milliseconds, plotted in gray columns of Figure 4A and 4B for comparison) it is actually larger than that effect size in units of standard deviations (E1 here, d = 0.835, B&F E1, d = 0.665). Accordingly, while this result is interpreted here as evidence only for a contribution of the CoM confounds to B&F’s findings, there is the potential for CoM to provide a complete account, as I discuss below.

### Conclusion

The current experiment used four pairs of stimuli that were (in broad outline) similar to (four of) Boger and Firestone’s ‘visual anagrams’ in the sense that, within each pair of stimuli, rotating them, changed their CoM. In B&F’s original stimuli, this rotation also elicited a striking change in the perceived category of object, whereas in the current experiment, this change was minimised. Accordingly, if the current experiments were to have shown broadly similar Stroop effects to those reported by B&F, it might have been concluded that these, and B&F’s effects, solely reflected changes in CoM. Conversely, if no Stroop effect was observed here, it might have be concluded that B&F’s findings had reflected purely categorisation of those shapes, as they intended.

It appears that there *is* a Stroop Effect with these new stimuli, even when trials with potential categorisation effects are removed. This is evidence that CoM confounds *did* contribute to B&F’s findings. The small raw effect size is 30–50% of those reported by B&F in milliseconds – consistent with only a partial contribution, but given that RTs here were rather faster than those in B&F’s studies, the smaller raw Stroop Effects here (in milliseconds) may simply reflect scaling of effects with mean RT. Indeed, in the primary analysis here, as mentioned earlier, the effect in standard deviations was larger (d = 0.835) than B&F’s Experiment 1 (d = 0.665). There remains, accordingly, potential for CoM effects to scale up with RTs; future research may address this. These possibilities aside, I conclude that CoM confounds contributed to B&F’s Familiar Size Stroop Effects but cannot currently account for the majority of the effect.

In this brief Methods Note, I have not distinguished between alternative senses of CoM that arise when an image depicts a familiar, three-dimensional object. In these cases, the CoM may reflect either the image’s 2D structure or the depicted-object’s known 3D structure. Similarly, it might be the *absolute* height of a shape’s CoM’s in the display relative to the other shape that generates an apparent Familiar Size Stroop task, or alternatively, the *relative* height of the CoM in the object (whether it is ‘top-heavy’ or ‘bottom-heavy’). These alternative possibilities are not distinguished here, but future work may need to do so.

This Methods Note is also, of course, limited in scope. As only a few stimuli were employed (necessary to parallel B&F’s work), the current findings cannot provide general evidence about low-level feature confounds in the Familiar Size Stroop Task. Neither can they support any general claims about visual anagrams. Rather, the current experiment speaks only to the potential role of *CoM confounds in B&F’s stimuli* when interpreting their Stroop Effects. Nonetheless, the current work does provide a single example of potential issues when using visual anagrams – one that should alert us to the potentially wider range of potential confounds alluded to in the Introduction.

In summary, Boger and Firestone’s use of visual anagrams to help control visual properties of stimuli when studying higher-level vision is likely a considerable advance and will be widely adopted. Nonetheless, rotating a stimulus only retains (and therefore controls) a subset of its visual properties. Other properties vary with rotation and we must be vigilant to ensure they do not yield effects we would attribute to higher-level processes. Here, Familiar Size Stroop Effects appeared to arise on the basis of properties that are not controlled by visual anagrams B&F employed. These outcomes are certainly not fatal for the adoption of visual anagrams. Rather, they highlight that rotating a stimulus *introduces* some new subtle confounds and that students of visual cognition need to be cognisant of these when using those techniques.

## Appendix

**Supplementary Table 1:** Labels entered by individual participants when asked whether each individual shape had reminded them of a specific real-world, familiar object (participants who reported no recognition are not included above, for brevity). A “>” symbol indicates where a decision was made that the label was a larger real-world object than the other version of that same visual anagram. Note, participants categorising shapes was considered problematic if they categorised the high CoM shape as a larger familiar object than the low CoM object (see text). Second, categorising only one of the two shapes in a pair was typically considered problematic because such an effect could not be ruled out (except in 2 cases when ‘tooth’ was judged ‘small’).

PARTICIPANT	ANAGRAM 1 HIGH CoM	ANAGRAM 1 LOW CoM	ANAGRAM 2 HIGH CoM	ANAGRAM 2 LOW CoM	ANAGRAM 3 HIGH CoM	ANAGRAM 3 LOW CoM	ANAGRAM 4 HIGH CoM	ANAGRAM 4 LOW CoM

1					Tooth	Tooth	Kiwi (Animal)	Kiwi (Animal)

2	tap	Duck >		chair	buffalo	buffalo	kiwi bird	

3	tap	Duck >			tooth	dog >	bird	bunny >

4	duck	duck			tooth with root	cat or rabbit >	bird	Rabbit >

5	Intestines			A country	Tooth	A bra >	A bird	Rabbit >

6		duck			tooth	cat >	rabbit	rabbit

10	tap	Duck >				dog	bird	rabbit >

11	Chair >	Duck			Tooth	Plastic bag >	Bird (kiwi)	Rabbit >

12		duck				cat	bird	rabbit >

15	tap	Duck >			molar	Dog >	bird	Rabbit >

16	Tap >	snail			teeth or cow	Puppy >		Rabbit >

17		duck			upside down america	Upside down america	bird	rabbit >

18		duck		book	tooth Tooth=Small		rabbit	rabbit

19	Duck	Duck			Elephant	Elephant	Kiwi	Rabbit >

20		duck			tooth	tooth	Swan >	rabbit

21		folded paper duck or swan			plastic bag upside down	plastic bag >	bird statue	Rabbit >

22		Duck			Tooth	Dog >	Bird	Rabbit >

23		Duck			Bison	Bison	Bird	Rabbit >

24	rubber duck	rubber duck			tooth	tooth	rabbit	rabbit

25		origami swan			Elephant >	cat	kiwi bird	Rabbit >

27		duck				dog		rabbit

28	Duck	Duck	The state of Missouri		Tooth (*small*)	Tooth (when it was shown the other way around)	Bird	Rabbit >

30	Chair >	duck			Elephant >	Tiny dog	bird of prey >	bunny

31		Duck		America	Cow	Cow	Bird	Bird
